# No Effect of Hypercholesterolemia on Elastase-Induced Experimental Abdominal Aortic Aneurysm Progression

**DOI:** 10.3390/biom11101434

**Published:** 2021-09-30

**Authors:** Toru Ikezoe, Takahiro Shoji, Jia Guo, Fanru Shen, Hong S. Lu, Alan Daugherty, Masao Nunokawa, Hiroshi Kubota, Masaaki Miyata, Baohui Xu, Ronald L. Dalman

**Affiliations:** 1 Department of Surgery, Stanford University School of Medicine, Stanford, CA 94305, USA; ikezoe@ks.kyorin-u.ac.jp (T.I.); dr.snow720@gmail.com (T.S.); guojia212@163.com (J.G.); sfr0907@163.com (F.S.); 2 Department of Cardiovascular Surgery, Kyorin University School of Medicine, Mitaka, Tokyo 181-8611, Japan; m_nunokawa@hotmail.com (M.N.); kub@ks.kyorin-u.ac.jp (H.K.); 3 Department of Emergency Medicine, Saiseikai Central Hospital, Minatoku, Tokyo 108-0073, Japan; 4 Saha Cardiovascular Research Center, Department of Physiology, University of Kentucky, Lexington, KY 40536, USA; hlu4@uky.edu (H.S.L.); alan.daugherty@uky.edu (A.D.); 5 Faculty of Medicine, School of Health Sciences, Kagoshima University, Kagoshima 890-8544, Japan; miyatam@m3.kufm.kagoshima-u.ac.jp

**Keywords:** abdominal aortic aneurysm, hypercholesterolemia, high-fat diet, PCSK9, leukocytes, angiogenesis

## Abstract

Objective: Epidemiological studies link hyperlipidemia with increased risk for abdominal aortic aneurysms (AAAs). However, the influence of lipid-lowering drugs statins on prevalence and progression of clinical and experimental AAAs varies between reports, engendering controversy on the association of hyperlipidemia with AAA disease. This study investigated the impact of hypercholesterolemia on elastase-induced experimental AAAs in mice. Methods: Both spontaneous (targeted deletion of apolipoprotein E) and induced mouse hypercholesterolemia models were employed. In male wild type (WT) C57BL/6J mice, hypercholesterolemia was induced via intraperitoneal injection of an adeno-associated virus (AAV) encoding a gain-of-function proprotein convertase subtilisin/kexin type 9 mutation (PCSK9) followed by the administration of a high-fat diet (HFD) (PCSK9+HFD) for two weeks. As normocholesterolemic controls for PCSK9+HFD mice, WT mice were infected with PCSK9 AAV and fed normal chow, or injected with phosphate-buffered saline alone and fed HFD chow. AAAs were induced in all mice by intra-aortic infusion of porcine pancreatic elastase and assessed by ultrasonography and histopathology. Results: In spontaneous hyper- and normo-cholesterolemic male mice, the aortic diameter enlarged at a constant rate from day 3 through day 14 following elastase infusion. AAAs, defined as a more than 50% diameter increase over baseline measurements, formed in all mice. AAA progression was more pronounced in male mice, with or without spontaneous hyperlipidemia. The extent of elastin degradation and smooth muscle cell depletion were similar in spontaneous hyper- (score 3.5 for elastin and 4.0 for smooth muscle) and normo- (both scores 4.0) cholesterolemic male mice. Aortic mural macrophage accumulation was also equivalent between the two groups. No differences were observed in aortic accumulation of CD4^+^ or CD8^+^ T cells, B cells, or mural angiogenesis between male spontaneous hyper- and normocholesterolemic mice. Similarly, no influence of spontaneous hypercholesterolemia on characteristic aneurysmal histopathology was noted in female mice. In confirmatory experiments, induced hypercholesterolemia also exerted no appreciable effect on AAA progression and histopathologies. Conclusion: This study demonstrated no recognizable impact of hypercholesterolemia on elastase-induced experimental AAA progression in both spontaneous and induced hypercholesterolemia mouse models. These results add further uncertainty to the controversy surrounding the efficacy of statin therapy in clinical AAA disease.

## 1. Introduction

Abdominal aortic aneurysms (AAAs) are a life-threatening consequence of aging. Development of AAA disease is predicated on both inherited and acquired predispositions (www.cdc.gov/heartdisease/aortic_aneurysm.htm, accessed on 20 September 2021). Epidemiological studies associate hypercholesterolemia with modest AAA disease risk (odds ratio 1.31–1.44) relative to those posed by cigarette smoking (2.61–12.13, depending on intensity and duration), male sex (5.71) and advancing age (1.67–28.37) [1,2]. More recently, elevated lipid variant genetic risk scores have also been associated with AAA disease (odds ratio 1.40–1.47) [3]. However, interventional trials of serum lipid-lowering pharmaceutical strategies to limit progression of clinical and experimental AAAs using β-hydroxy β-methylglutaryl-CoA reductase inhibitors, aka statins, have produced inconsistent results [4,5,6,7,8,9,10,11,12,13].

More than 60% of mice with genetic and diet-induced hypercholesterolemia develop AAAs following subcutaneous angiotensin (Ang) II administration [13,14,15,16]. Given the dependency on exogenous Ang II for aneurysm formation in these models, however, the influence of hypercholesterolemia as an independent risk factor for experimental AAA progression has been difficult to assess.

In the present study, the influence of spontaneous and induced hypercholesterolemia on experimental AAA progression was evaluated in an alternative model, with aneurysms created by transient intra-aortic infusion of porcine pancreatic elastase (PPE). This model demonstrates progressive aneurysmal aortic enlargement and multiple histopathologic features characteristic of clinical aneurysm disease without a prerequisite for hypercholesterolemia at AAA initiation. This enabled an independent assessment of the influence hypercholesterolemia on experimental AAA pathogenesis.

## 2. Materials and Methods

### 2.1. Induction of Hypercholesterolemia and Definition of Model-Specific Control Mice

Two complementary mouse hypercholesterolemic modeling systems were employed. ApoE^−/−^ mice on a C57BL/6J genetic background served a spontaneous hypercholesterolemia model, with wild type (WT) C57BL/6J mice serving as normocholesterolemic controls. In a second model, male WT C57BL/6J mice were infected with proprotein convertase subtilisin/kexin type 9 (PCSK9) D377Y adeno-associated virus (AAV) via i.p. (intraperitoneal) injection. This AAV construct encodes a gain-of-function (GoF) mutation to PSCK9 to degrade low-density lipoprotein (LDL) receptor function in infected cells. These mice were subsequently fed high-fat-diet (HFD) chow (42% calories from fat, TD.88137, ENVIGO, Indianapolis, IN, USA) exclusively for two weeks prior to AAA initiation (PCSK9+HFD group) [17]. As normocholesterolemic controls for PCSK9+HFD mice, WT mice were either infected with PCSK9 AAV and fed a normal laboratory diet or injected with phosphate-buffered saline (PBS) alone and fed the HFD. Serum cholesterol concentrations were measured using PRIMA cholesterol test strips (Prima Lab SA, Mano, Switzerland).

All mice were obtained from The Jackson Laboratory (Bar Harbor, ME), and used at the age of 8–9 weeks for experiments. Animal use, care, and experimental procedures in this study were approved by the Stanford Administrative Panel on Animal Care (APLC protocol #11131) and conducted in compliance with the Stanford Laboratory Animal Care Guidelines.

### 2.2. Experimental AAA Creation

Aneurysms were created by transient intra-infrarenal aortic infusion of PPE as detailed previously [13,14]. Briefly, the mice were anesthetized with isoflurane inhalation, and all surgical procedures were performed under sterile conditions. The infrarenal aorta was exposed and controlled via midline laparotomy, with ligation of all visualized branch arteries. The aortic bifurcation was cannulated with BTPU-010 polyurethane tube to allow the infusion of 30 μL PPE (1.5 U/mL in saline) over 5 min. In 8–10-week-old WT C57BL/6J normocholesteroemic male mice, this infusion procedure is sufficient for producing AAAs with an average maximal luminal aortic diameter of 1.2–1.3 mm within 14 days following PPE infusion. The aortotomy was closed with 10–0 nylon suture. Following closure of the laparotomy, mice were housed in individual cages for postsurgical recovery and aneurysm monitoring. The surgical mortality in this model ranges from 10 to 15%.

### 2.3. Imaging AAA Formation and Progression

Aortic aneurysmal enlargement was monitored by serial transabdominal high frequency ultrasound (40 MHz, Vevo 2100, VisualSonics, Toronto, ON, Canada) assessments following postsurgical recovery [13]. Measurements were performed one day prior to (baseline, day 0), and 3, 7, and 14 days following, PPE infusion by investigators blinded to experiment group assignment. In each ultrasound examination, the maximal internal aortic diameter within the PPE-infused infrarenal segment was measured from transverse aortic images stored within the Vevo@Imaging System software. The presence of AAA was defined as a ≥ 50% increase in pre-infusion aortic diameter. Although definitions vary as to what degree of aortic diameter constitutes an “aneurysm”, in this model, a ≥ 50% increase compared to age-adjusted population norms is consistent with the clinical definition of AAA disease. In PBS-infused control mice, the magnitude of infusion-induced mechanical distension of the infrarenal segment in the absence of aneurysm formation was reflected by average diameter enlargements of 21%, 18% and 15% on days 3, 7 and 14, respectively. 

### 2.4. Histological Analyses

**Figure 1:**
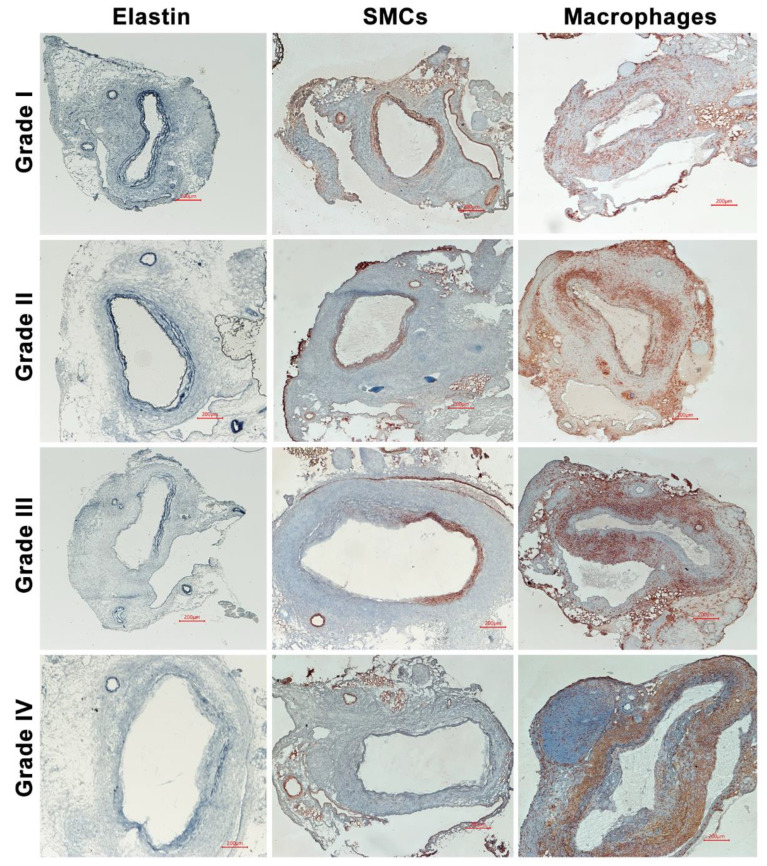
Representative histological images demonstrating the grading system used to categorize medial elastin and SMC destruction and macrophage accumulation. Aneurysmal aortas from porcine pancreatic elastase-infused mice were stained with Elastic Van Gieson (EVG) stain for medial elastin, SMC α-actin antibody for SMCs and CD68 antibody for macrophages, respectively. Medial elastolysis and SMCs as well as macrophage densities were graded as I (mild) to IV (severe). Grade I: elastin degradation, SMC loss, or aggregate macrophage accumulation involves <25% of aortic circumference (AC) or diffuse macrophage infiltration. Grade II: elastin degradation, SMC loss, or aggregate macrophage accumulation involves ≥25% but <50% of AC. Grade III: elastin degradation, SMC loss, or aggregate macrophage accumulation involves ≥50% but <75% of AC. Grade IV: elastin degradation, SMC loss, or aggregate macrophage accumulation involves ≥75% of AC.

Mice were sacrificed 14 days after PPE infusion. Aortae were harvested, embedded in optimal cutting temperature compound media, sectioned (6 μm), and acetone-fixed. Medial elastin was assessed using Elastic Van Gieson (EVG) stain [18]. A 2- or 3-step standard immunoperoxidase procedure for immunohistochemistry was used to identify medial smooth muscle cells (SMC, SMC α-actin), mural neoangiogenesis (CD31) and aortic leukocyte accumulation (macrophages, CD4 and CD8 T cells, and B cells) using subset-specific monoclonal antibodies (mAbs). Primary and secondary antibodies, other immunostaining reagents, and their working conditions are provided in Table 1. Based on staining patterns of EVG, SMC α-actin mAb, or CD68 mAb, medial elastin degradation, SMC depletion, and macrophage accumulation were scored on a scale of I (mild) to IV (severe) (Figure 1) [19]. Mural neoangiogenesis and other leukocytes were quantified as CD31-positive vessels or subset mAb-positive cells per aortic cross-section (ACS).

### 2.5. Statistical Analyses

All statistical analyses were performed using GraphPad Prism (Ver 8.0.1 GraphPad Prism Software, Inc., San Diego, CA, USA). The Shapiro–Wilk normality test was used to determine whether individual datasets were normally distributed. All normally distributed data were presented as mean and 95% confidence interval (CI), and either Student’s *t*-test or two-way repeated measures ANOVA, followed by the Newman–Keuls post hoc test, were used to determine the significance between groups. All non-normally distributed data are presented as median and 95% CI, and nonparametric Mann–Whitney test was used to determine differences between two groups. In all statistical analyses, *p*<0.05 was considered significant.

## 3. Result

### 3.1. No Effect of Spontaneous Hypercholesterolemia on Experimental AAA Diameter

**Figure 2:**
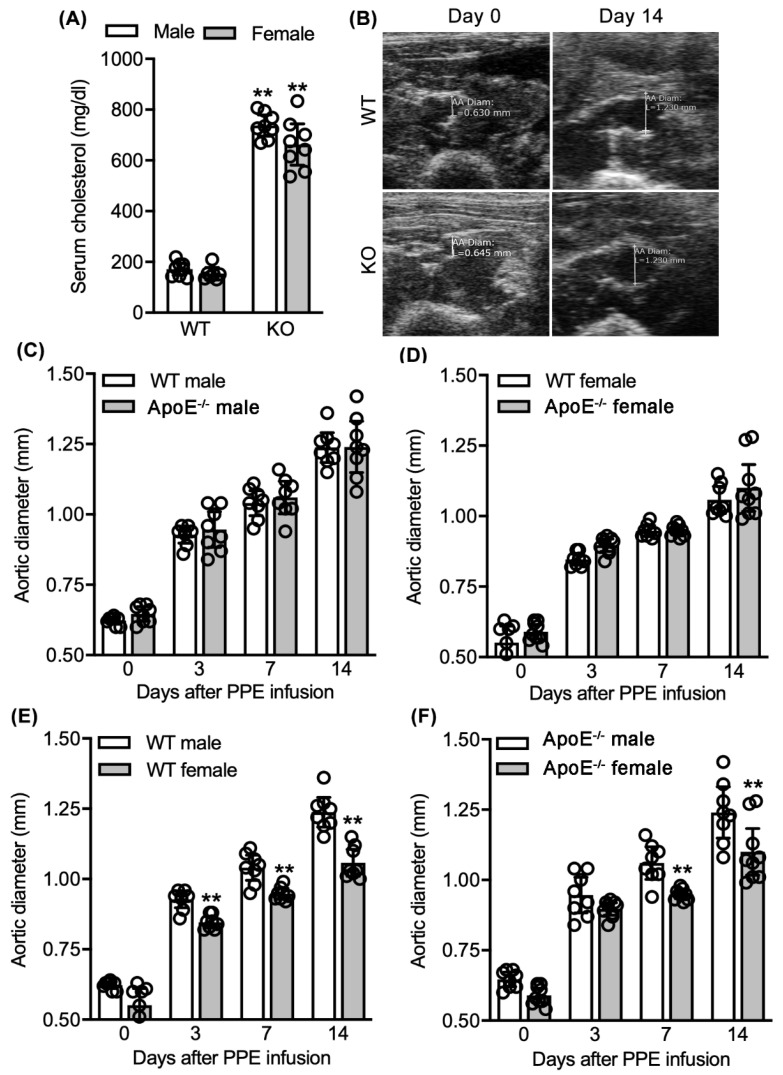
Influence of spontaneous hypercholesterolemia on elastase-induced experimental AAAs. Following intra-aortic porcine pancreatic elastase (PPE) infusion, normo- (WT) and hypercholesterolemic ApoE^−/−^ mice were monitored for AAA formation and progression via serial ultrasound measurements of maximal infrarenal luminal diameters. (**A**) Mean and 95% confidence interval (CI) of serum cholesterol concentrations in WT and ApoE^−/−^ mice measured via cholesterol strips. (**B**) Representative aortic ultrasound images prior to, and 14 days after, PPE infusion in male WT and ApoE^−/−^ mice. (**C**,**D**) Mean and 95% CI of maximal infrarenal aortic diameters in male (**C**) and female (**D**) WT and ApoE^−/−^ mice at indicated intervals. (**E**) Presence of sex difference in aneurysmal aortic diameter in WT male and female mice. Mean and 95% CI of maximal infrarenal aortic diameters in WT male and female mice prior to, and indicated days after, PPE infusion. (**F**) Presence of sex difference in aneurysmal aortic diameter in ApoE^−/−^ male and female mice. Mean and 95% CI of maximal infrarenal aortic diameters in ApoE^−/−^ male and female mice prior to, and indicated days after, PPE infusion. In A, Student’s *t*-test, ** *p* < 0.01 compared to male or female WT mice; In (**E**,**F**), two-way ANOVA followed by Newman–Keuls post hoc test, ** *p* < 0.01 compared with WT male or female mice at same timepoints. *n* = 8 for male WT and ApoE^−/−^ mice; *n* = 7 and 8 for female WT and ApoE^−/−^ mice, respectively.

Both male (mean and 95% CI: 737, 695–779 mg/dL) and female (662, 580–744 mg/dL) ApoE^−/−^ mice achieved significantly higher serum cholesterol concentrations than those measured in age-matched WT male (171, 147–195 mg/dL) and female (154, 129–178 mg/dL) mice (Figure 2A).

Following PPE infusion, both male WT and ApoE^−/−^ mice experienced significant infrarenal aortic enlargement from day 3 through day 14 (Figure 2B,C). The rate of enlargement did not differ between the two groups at any time following AAA induction. Aneurysm diameters were 1.4 (1.18–1.29) (mean and 95% CI) and 1.24 (1.15–1.33) mm for WT and ApoE^−/−^ mice, respectively, 14 days following PPE infusion. Similar progression was noted in female mice (Figure 2D). Regardless of sex or strain, all mice developed AAAs as defined by a more than 50% increase in aortic diameter over baseline measurements.

Additionally, female mice experienced less aortic diameter enlargement than strain-matched male mice following PPE infusion, regardless of ApoE status (Figure 2E,F). These data indicate that spontaneous hypercholesteremia due to ApoE deficiency had no impact on sex-dependent differences in experimental AAA progression. Taken together, these results indicate that spontaneous hypercholesterolemia in mice deficient for ApoE exerts minimal influence on development and progression of PPE-induced experimental AAAs.

### 3.2. No Effect of Spontaneous Hypercholesterolemia on AAA Histopathology

The potential influence of hypercholesterolemia on characteristic experimental AAA histopathology was assessed by comparison of aortic medial elastin degradation, SMC depletion, mural leukocye accumulation, and neovessel formation between groups.

**Figure 3:**
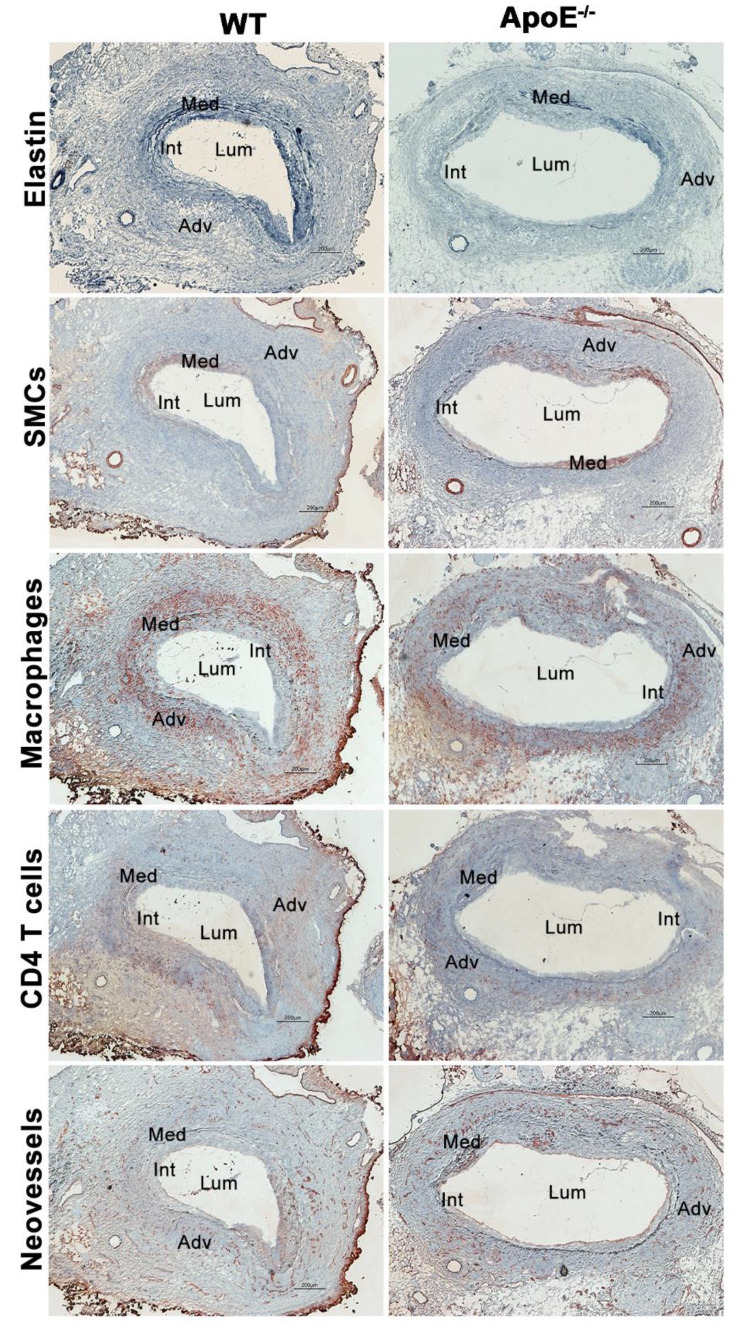
Representative AAA histology in male normocholesterolemic WT and spontaneous hypercholesterolemic ApoE^−/−^ mice. Fourteen days following elastase infusion, aortae were harvested, embedded in optimal cutting temperature compound, sectioned (6–8 μm), and fixed with acetone. Medial elastin was stained with Elastic Van Gieson (EVG) staining. Smooth muscle cells (SMC), macrophages, CD4-positive T cells, and neovessels were stained using monoclonal antibodies to SMC α-actin, CD68, CD4, and CD31. Lum: lumen. Int: Intima. Med: Media. Adv: Adventitia. Scale bar: 200 μm.

**Figure 4:**
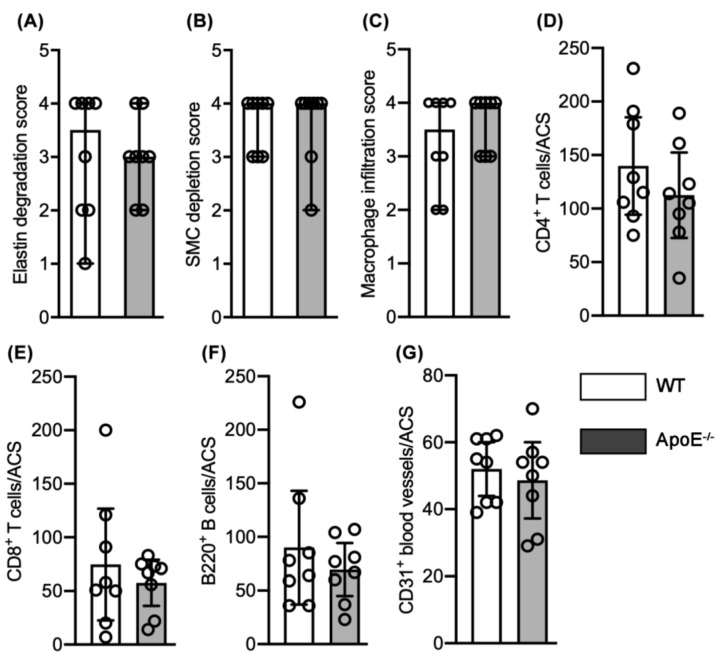
Quantification of AAA histopathological features between elastase-infused male normocholesterolemic WT and hypercholsterolemic ApoE^−/−^ mice (*n* = 8 mice/group). (**A**–**C**) Median and 95% confidence interval (CI) of the scores for medial elastin (**A**) and smooth muscle cell (SMC) (**B**) destruction as well as CD68-positive macrophage accumulation. Score I to IV represent mild to severe destruction. (**D**,**E**) Mean and 95% CI for CD4^+^ T cells (**E**) and CD8^+^ T cells (**F**) per aortic cross-section (ACS). (**F**) Median and 95% CI for B220-positive B cells per ACS. (**G**) Mean and 95% CI of CD31-positive neovessels per ACS. Nonparametric Mann–Whitney test (**A**–**C**,**F**) or Student’s *t*-test (**D**,**E**,**G**) indicated no significance difference between two groups.

Elastin degradation was scored as 3.5 (1.0–4.0) (median and 95% CI) in male normocholesterolemic WT mice, involving 50 to 75% of the aortic circumference (AC) (Figure 3 and Figure 4A). This degree of elastolysis was not significantly different from that noted in hypercholesteremic ApoE^−/−^ male mice (3.0, 2.0–4.0). SMC depletion was graded as 4.0 (3.0–4.0) and 4.0 (2.0–4.0) for male normo- and hypercholesterolemic mice, respectively, involving more than 75% of AC (Figure 3 and Figure 4B). Again, no difference was noted between groups.

Macrophages were the predominant leukocyte subset present in aneurysmal lesions from both normo- and hyper-cholesterolemic male mice (Figure 3 and Figure 4). The median score for macrophage accumulation was 3.5 (2.0–4.0) in male normocholesterolemic mice, indicating macrophage accumulation in 50–75% of AC. Such accumulation was similar to those noted in male hypercholesterolemic mice (4.0, 3.0–4.0; Figure 3 and Figure 4C). Normocholesterolemic male mouse aortae demonstrated more aortic mural CD4 (mean and 95% CI: 140, 94–185 cells/ACS) and CD8 (74, 23–127 cells/ACS) positive T cells as compared to hypercholesterolemic controls (113, 73–152 cells/ACS for CD4 T cells and 58, 36–79 cells/ACS for CD8 T cells) (Figure 3 and Figure 4D,E). B cells accumulated at the same magnitude in both male normo- (media and 95% CI: 71, 36–226) and hypercholesterolemic (72, 23–107) mice (Figure 3 and Figure 4F). Overall, no significant differences in individual leukocyte subsets were noted between normo- and hyper-cholesterolemic male mice.

Mural neoangiogenesis is another hallmark pathologic feature of aneurysmal aortic degeneration. Neovessel density, as enumerated by CD31-positive vessels, was indistinguishable between normo- (mean and 95% CI: 55, 39–62 vessels/ACS) and hypercholesterolemic (52, 29–70 vessels/ACS) mouse aortae (Figure 3 and Figure 4G).

**Figure 5:**
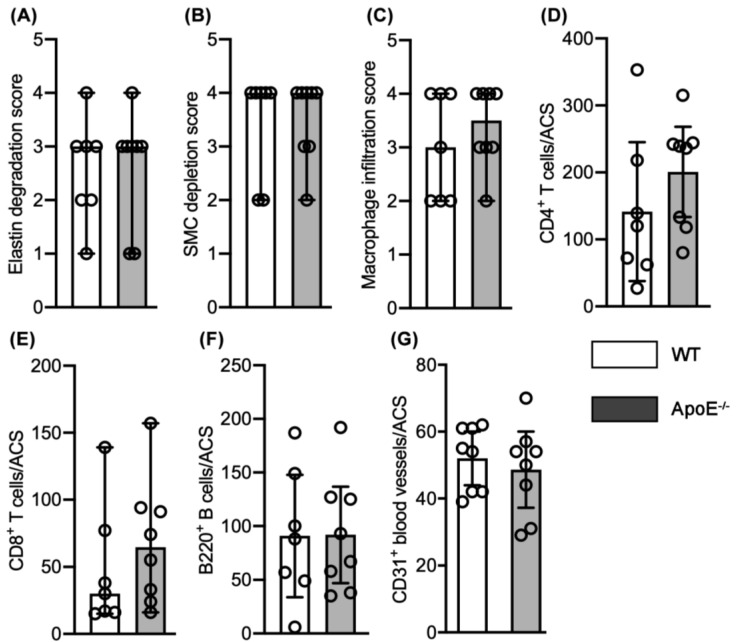
Quantification of AAA histopathological features in elastase-infused normocholesterolemic WT and hypercholesterolemic ApoE^−/−^ female mice (*n* = 7 and 8 mice in WT and ApoE^−/−^ mouse groups, respectively). (**A**–**C**): Media and 95% confidence interval (CI) of the scores for medial elastin (**A**) and smooth muscle cell (SMC) (**B**) destruction as well as CD68-positive macrophage accumulation (**C**). Scores I to IV represent mild to severe destruction. (**D**): Mean and 95% CI for CD4^+^ T cells (**E**) per aortic cross-section (ACS). (**E**,**F**): Media and 95% CI for CD8^+^ T cells (**E**) B220^+^ B cells (**F**) per ACS. (**G**): Mean and 95% CI of CD31^+^ neovessels per ACS. Nonparametric Mann–Whitney test (**A**–**C**,**E**,**F**) or Student’s *t*-test (**D**,**G**) indicated no significance difference between two groups.

Similar histological analyses were also performed for aortic sections from PPE-infused female normo- and hypercholesterolemic mice. Median scores for elastin and SMC destruction were identical, scoring as 4.0 with 95% CI of 1.0–4.0 (EVG) and 2.0–4.0 (SMC) (Figure 5A,B). While more macrophages, CD4^+^ T cells and CD8^+^ T cells were noted in hyper- as compared to normocholesterolemic female mouse AAAs, these differences were not statistically significant (Figure 5C–E). Likewise, the extent of B cell accumulation and aortic mural angiogenesis were indistinguishable between groups (Figure 5F,G).

These results indicate that spontaneous hypercholesterolemia exerted no measurable effect on characteristic experimental AAA histopathology induced by intra-aortic transient PPE infusion.

### 3.3. No Differences in Aneurysmal Progresssion and Histopathologies Noted between Normocholesterolemia and “Acquired” Hypercholesterolemia

**Figure 6:**
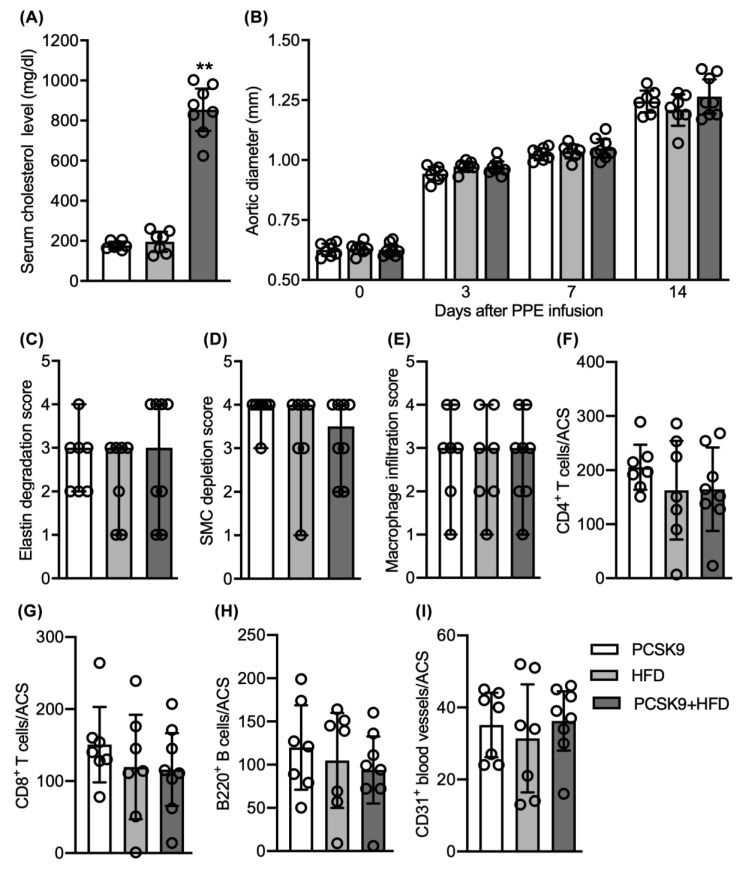
Influence of induced hypercholesterolemia on aneurysm enlargement and aortic pathologies. Male WT mice at age of 8 weeks were injected with phosphate-buffered saline (PBS) and immediately fed a high-fat diet (HFD), infected with PCSK9/AAV and immediately fed either normal laboratory chow (PCSK9) or HFD (PCSK9+HFD). Two weeks thereafter, mice underwent transient intra-aortic PPE infusion for AAA induction and continued on their pre-assigned diets. (**A**) Mean and 95% confidence interval (CI) of serum cholesterol concentrations measured via cholesterol strips (*n* = 7 mice in PCSK9 and HFD groups and 8 mice in PCSK9+HFD group). (**B**) Mean and 95% CI of infrarenal aortic luminal diameters prior to and indicated days after, PPE infusion imaged via ultrasonography. (**C**–**E**) Median and 95% CI for histological scores for elastin degradation (**C**), smooth muscle cell (SMC) depletion (**D**) and macrophage (**E**). Medial elastin degradation, SMC depletion and macrophage accumulation were graded as I (mild) to IV (severe). (**F**–**I**) Mean and 95% CI of CD4^+^ T cells (**F**), CD8^+^ T cells (**G**), B220^+^ B cells (**H**) and CD31^+^ neovessels (**I**) per aortic cross-section (ACS). Student’s *t*-test, ** *p* < 0.01 compared to PCSK9 and HFD groups (**A**). Two-way ANOVA followed by Newman–Keuls post hoc test, no difference was noted between the groups (**B**). Nonparametric Mann–Whitney test indicated no significant difference between two groups, test (**B**–**I**).

To validate and generalize these findings beyond those observed in spontaneous hypercholesterolemia, an “acquired” mouse hypercholesterolemia model was developed and employed. In this model, male WT C57BL/6J mice were intraperitoneally injected with an AAV encoding GoF mutation of PCSK9 and subsequently fed a HFD for 2 weeks (PSCK9+HFD). These interventions resulted in significant elevation of serum cholesterol concentrations in PCSK9+HFD mice (mean and 95% CI: 854, 749–959 mg/dL) as compared to mice inoculated with PCSK9 AAV and fed a normal laboratory diet alone (177, 159–196 mg/dL) or injected with PBS alone and fed a HFD (196, 146–246). This confirmed the successful induction of hypercholesterolemia in this model (Figure 6A).

All mice experienced equal time-dependent, progressive aortic enlargement following PPE infusion regardless of GoF PCSK9 or dietary category (Figure 6B) in these experiments. On day 14, aortic diameters were 1.21 (1.14–1.27) (mean and 95% CI), 1.27 (1.19–1.34) and 1.24 (1.19, 1,29) mm in HFD, PCSK9 and PCSK9+HFD mice, respectively. AAAs developed in all mice within 7 days following PPE infusion.

No significant differences were found for medial elastin and SMC destruction among the groups, with median scores of 3.0 for elastin degradation and 3.5 to 4.0 for SMC depletion (Figure 6C,D). Induced hypercholesterolemia also did not affect mural macrophage accumulation with all groups scoring 3 with a 95% CI of 1.0 and 4.0, indicating dense focal macrophage accumulation involving 50 to 75% of the aortic circumference (Figure 6E). Mural accumulation of CD4^+^ T cells, CD8^+^ T cells, and B cells, as evaluated by positively stained cells/ACS, were not significant for any leukocyte subsets (Figure 6F–H). Similarly, neovessel density, as quantified by CD31^+^ vessels per ACS, was indistinguishable among three groups (Figure 6I).

Collectively, these results support the conclusion that hypercholesterolemia, whether spontaneous or HFD-induced, does not influence progression of experimental AAAs induced via intra-aortic PPE infusion. 

## 4. Discussion

In the PPE intra-aortic infusion model, the presence of hypercholesterolemia exerted no significant effect on the prevalence, progression, or histopathological features of experimental AAAs. These findings support those of Steinmetz and associates, who found no effect of ApoE deficiency on experimental AAAs created in a similar modeling system [20]. In alternative AAA modeling systems, however, hypercholesterolemic ApoE^−/−^ mice reportedly experienced more significant experimental aneurysm progression [21], challenging the translational relevance of findings from both models. However, significant differences exist in the mechanisms and consequences of AAA induction between models. In the latter case, following AAA induction via abluminal application of calcium chloride the ApoE^−/−^ mice were noted to have more pronounced aortic medial calcification, creating the possibility that calcification, rather than hypercholesterolemia per se, may accelerate experimental AAA progression [22,23,24,25,26]. 

Prior studies evaluating the ability of lipid-lowering drugs to suppress experimental aneurysms in distinct AAA modeling systems have yielded inconsistent results. For instance, simvastatin, across a large dose range (2–60 mg/kg), inhibited the progression of experimental AAAs created by intra-aortic elastase infusion, subcutaneous Ang II infusion, or abluminal peri-aortic calcium chloride application without apparent influence on serum cholesterol concentrations [10,11,20,27,28,29]. Pitavastatin mitigated experimental AAAs induced by either elastase infusion in normocholesterolemic rats, or Ang II infusion in hypercholesterolemic mice, in conjunction with enhanced intracellular drug concentrations resulting from nanoparticle-enhanced delivery, also without apparent effect on serum cholesterol concentrations [30,31]. Atorvastatin also suppressed experimental AAAs at high (20 mg/kg) but not intermediate (10 mg/kg) or low (1 mg/kg) doses, also without modulating circulating cholesterol concentrations. However, fluvastatin or rosuvastatin administration, although effective in attenuating expression of certain aortic inflammatory mediators, did not influence the incidence, enlargement, or rupture of experimental AAAs. From these studies, it is apparent that when present, statin-mediated experimental AAA suppression may result from off-target pharmacological effects unrelated to cholesterol-lowering effect or cholesterol metabolism. Regardless of the efficacy of specific statins or the mode of administration, these findings add credence to the conclusion that hypercholesterolemia alone does not promote or accelerate experimental AAA progression.

Ang II and its type 1 receptor mediate AAA pathogenesis in both exogenous Ang II-dependent and independent models [13,32,33,34]. In normocholesteroemic mice, minimal AAA formation was noted in response to exogenous Ang II administration [35]. Hypercholesterolemia, regardless of the method of induction, significantly enhances the AAA-promoting effects of Ang II [15,16,17,36,37]. Beyond hypercholesterolemia, various factors also promote AAA formation following exogenous Ang II administration. These include obesity (as a consequence of long-term HFD) in the absence of hypercholesterolemia, neutralization of transforming growth factor-β, chemical destabilization of extracellular matrix protein cross-linking via β-aminopropionitrile monofumarate administration, and genetic deficiency of nuclear factor erythroid 2-related factor 2 [35,38,39,40,41,42]. Thus, while clearly capable of promoting experimental AAAs, hypercholesterolemia may not be a specific, absolute requirement for aneurysm induction and progression in response to exogenous Ang II.

Hypercholesterolemia is recognized as a mild to moderate epidemiologic risk for AAA disease relative to those posed by either cigarette smoking, male sex, or advanced age [1,2]. Statin therapy also reduces all-cause short- and long-term mortality following surgical AAA repair [7,43,44]. Controversy continues regarding the influence of statin therapy on disease progression. This is partly due to lingering uncertainty regarding the value of the rate of AAA enlargement as a valid clinical endpoint vs. the “harder” endpoints of surgical repair, rupture, or aneurysm-related death. Statins have shown efficacy in limiting further AAA enlargement in small cohort prospective studies or meta-analyses combining cross-sectional, retrospective, and prospective studies without subgroup analyses [4,5,6,7]. Almost uniformly, these preliminary reports have not been validated by larger prospective studies or high-quality subgroup meta-analyses [8,9,45]. The experimental finding that hypercholesterolemia exerted no independent effect on experimental AAA initiation or progression adds further perspective to the inconsistent clinical observations regarding statin therapy and AAA disease progression.

PCSK9 lowers blood LDL cholesterol by binding to the cell surface LDL receptor. PCSK9 activity may be altered genetically or pharmacologically. In the Million Veterans Program of the United States Department of Veterans Affairs, a PCSK9 mutation associated with reduced serum LDL cholesterol levels was also associated with a 28% reduction in risk for AAA disease. In contrast, an enhanced polygenetic lipid variant risk score increased AAA risk by 40% or more [3]. In the current GoF mutant PCSK9 experiments, AAV infection alone, with or without a HFD, exerted no recognizable influence on experimental aneurysm progression or characteristic pro-aneurysmal aortic pathologies. Putative associations between PCSK9 variants or polygenetic lipid variant risk scores and AAA disease may be confounded by common risk factors for atherosclerosis and AAAs [46], or alterations in aortic inflammation, stiffness or structural features such as vascular-associated lymphoid tissues secondary to co-existing atherosclerosis may also link cholesterol to AAA risk [22,23,24,25,26,47,48,49]. Further analysis of AAA enlargement rates in patients treated with PCSK9 inhibitors (Alirocumab and Evolocumab) may provide further understanding of the role of serum lipid and cholesterol concentrations in clinical AAA disease.

## 5. Conclusions

These results provide compelling evidence that neither spontaneous nor induced hypercholesterolemia influences the initiation, enlargement, or characteristic pathologic progression of elastase-induced experimental AAAs in mice.

## Figures and Tables

**Table 1:** Sources and working conditions for immunohistochemical reagents.

Reagent	Manufacture	Catalog #	Clone #	Working Solution (μg/mL)	Incubation Time at Room Temperature (Minutes)
Rat anti-mouse-B220 mAb	Biolegend	103201	RA3-6B2	2.5	60
Rat-anti-mouse CD4 mAb	Biolegend	100402	GK1.5	2.5	60
Rat anti-mouse CD8 mAb	Biolegend	100702	53–6.7	2.5	60
Rat anti-mouse CD68 mAb	Biolegend	137002	FA-11	2.5	60
Biotin-mouse anti-mouse SMC α actin mAb	Thermo Fisher Scientific	MA5-11544	1A4	1.0	60
Biotin-goat anti-rat IgG antibody	Jackson ImmunoResearch Laboratories, Inc.	112-065-062	N/A	4.0	30
Peroxidase-streptavidin conjugate	Jackson ImmunoResearch Laboratories, Inc.	016-030-084	N/A	5.0	30
AEC peroxidase substrate Kit	Vector Laboratories, Inc.	SK-4200	N/A	N/A	10–20

mAb: monoclonal antibody.

## Data Availability

All data were included within this article.

## Abbreviation

AAA: abdominal aortic aneurysm; AAV: adeno-associated virus; AC: aortic circuference; ACS: aortic cross-section; Ang: angiotensin; ApoE: apolipoprotein E; CI: confidence interval; EVG: Elastic Van Gieson; GoF: gain-of-function; HFD: high-fat diet; i.p.: intra-peritoneal; LDL: low-density lipoprotein; mAb: monoclonal antibody; phosphate-buffered saline: PBS; PCSK9: proprotein convertase subtilisin/kexin type9; PPE: porcine pancreatic elastase; SMC: smooth muscle cell; WT: wild type.
